# Risk factors of peritoneal dialysis–related peritonitis in the Japan Peritoneal Dialysis Outcomes and Practice Patterns Study (PDOPPS)

**DOI:** 10.1093/ckj/sfae202

**Published:** 2024-07-01

**Authors:** Yasuhiko Ito, Charlotte Tu, Makoto Yamaguchi, Shigehisa Koide, Munekazu Ryuzaki, Brian Bieber, Ronald L Pisoni, Jeffrey Perl, Jun Minakuchi, Hideki Kawanishi, the Japan PDOPPS Study Committee, Hideki Kawanishi, Jun Minakuchi, Tadashi Tomo, Ken Tsuchiya, Kousaku Nitta, Munekazu Ryuzaki, Mizuya Fukazawa, Yasuhiro Ito, Hidetomo Nakamoto, Akihiro Yamashita

**Affiliations:** D epartment of Nephrology and Rheumatology, Aichi Medical University, Nagakute, Japan; Arbor Research Collaborative for Health, Ann Arbor, MI, USA; D epartment of Nephrology and Rheumatology, Aichi Medical University, Nagakute, Japan; Fujita Health University, Toyoake, Japan; Saiseikai Chuou Hospital, Tokyo, Japan; Arbor Research Collaborative for Health, Ann Arbor, MI, USA; Arbor Research Collaborative for Health, Ann Arbor, MI, USA; St. Michael's Hospital, Toronto, ON, Canada; Kawashima Hospital, Tokushima, Japan; Tsuchiya General Hospital, Hiroshima, Japan; PDOPPS steering committee member

**Keywords:** antibiotic prophylaxis, PDOPPS, peritoneal dialysis, peritonitis, risk factor

## Abstract

**Background:**

Peritoneal dialysis (PD)-related peritonitis is a major complication of PD. Wide variations in peritonitis prevention, treatment strategies and consequences are seen between countries. These between-country differences may result from modifiable risk factors and clinical practices.

**Methods:**

A total of 1225 Japanese PD patients were included and prospectively followed in the Peritoneal Dialysis Outcomes and Practice Patterns Study phase 1 (2014–2018) and phase 2 (2018–2022). Associations between PD-related peritonitis and various risk factors were assessed by Cox proportional hazards survival models.

**Results:**

During follow-up (median 1.52 years), 539 peritonitis episodes were experienced by 364 patients. The country crude peritonitis rate was 0.27 episodes/patient-year. In the fully adjusted model, noticeable patient-level factors associated with experiencing any peritonitis included age {hazard ratio [HR] 1.07 per 5-year increase [95% confidence interval (CI) 1.01–1.14]}, serum albumin level [HR 0.63 per 1 g/dl higher (95% CI 0.48–0.82)] and continuous ambulatory peritoneal dialysis (PD) [HR 1.31 versus automated PD (95% CI 1.05–1.63)]. The adoption of antibiotic prophylaxis practice at the time of PD catheter insertion [HR 0.63 (95% CI 0.51–0.78)] or when having complicated dental procedures [HR 0.74 (95% CI 0.57–0.95)] or lower endoscopy [HR 0.69 (95% CI 0.54–0.89)] were associated with lower hazards of any peritonitis, while a routine facility practice of having more frequent regular medical visits was associated with a higher hazard.

**Conclusion:**

Identification of risk factors in Japan may be useful for developing future versions of guidelines and improving clinical practices in Japan. Investigation of country-level risk factors for PD-related peritonitis is useful for developing and implementing local peritonitis prevention and treatment strategies

## Graphical Abstract

**Graphical Abstract:**
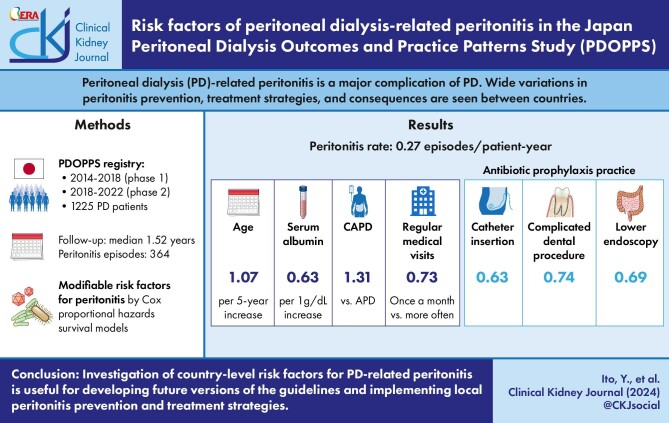

KEY LEARNING POINTS **What was known:**

- Peritoneal dialysis (PD)-related peritonitis is a major complication of PD, a major cause of withdrawal from PD, an important cause of death and varies considerably internationally along with prevention and treatment strategies.
- It is important to identify country-specific PD peritonitis risk factors, here focusing on Japan and its special context.

**This study adds:**

- Automated PD use, early PD education and antibiotic prophylaxis during PD catheter insertion, dental procedures and lower endoscopy were associated with lower peritonitis risks. However, peritonitis risk factors from other studies (hypokalaemia, constipation, gastric acid suppressant use, diabetes, cardiovascular diseases, high BMI) were not associated with peritonitis in Japan.

**Potential impact:**

- Identifying specific risk factors for PD-related peritonitis within a country may prove useful for establishing country-specific countermeasures, modifying future PD guidelines and improving clinical practices nationwide. These strategies may reduce the nationwide incidence of PD-related complications, improve the prognosis of PD patients and ultimately reduce healthcare costs.

## INTRODUCTION

Peritoneal dialysis (PD)-related peritonitis represents a major complication of PD. The Peritoneal Dialysis Outcomes and Practice Patterns Study (PDOPPS) enrolled nationally representative samples of PD patients from seven countries as a prospective cohort study. These studies examined the incidence of peritonitis, characteristics of causative organisms for peritonitis and exit site infections, differences in prophylaxis and treatment practices and the results of treatment and cure rate [1–4]. These studies found wide variations in PD-related infections, infection prevention practices, treatment strategies and outcomes [1–4].

The PDOPPS and other studies have investigated the optimal PD training program (examining how, how long, where, when and by whom training should be conducted), but the International Society for Peritoneal Dialysis (ISPD) guidelines indicate that no optimal training standards have yet been determined [5]. Difficulties in identifying the optimal training strategies from the PDOPPS may be due to country-specific differences in practices relating to bag exchange systems, the roles of nurses, education styles and culture. Various risk factors for PD-related peritonitis have been identified and reported, but no reports appear to have screened and identified risk factors for PD-related peritonitis from a comprehensive point of view in a single country.

The characteristics of PD in Japan include low penetration, small PD centre size, high use of continuous ambulatory peritoneal dialysis (CAPD) and the use of combined haemodialysis (HD)–PD hybrid therapy. Therefore, it is important to identify the problems based on the factors specific to the country and individual facilities to improve the quality of PD practice in Japan.

The aim of the present study was to explore the patient characteristics, dialysis facility characteristics and treatment practices associated with the risk of peritonitis and to identify country-specific risk factors for peritonitis in Japan.

## MATERIALS AND METHODS

### Data source

The PDOPPS is an international prospective cohort study investigating practices related to optimal outcomes for PD patients in Australia and New Zealand, Canada, Japan, South Korea, Thailand, the UK and the USA. Adult patients receiving maintenance PD (excluding hybrid therapy) were randomly selected from stratified random national samples of PD facilities treating at least 10 PD patients at the time of selection. Details of the study design and protocol have been published previously [6]. The study was approved by a central national or institutional review board, along with national and/or local ethics committees, as required by local ethics regulations. Written informed consent was obtained from all patients eligible for study participation. The present analysis was restricted to PDOPPS phase 1 (2014–2018) and phase 2 (2018–2022) in Japan. Japan PDOPPS phase 2 was largely a continuation of Japan PDOPPS phase 1 and the authors do not expect any major differences between the two phases in terms of protocol or facility/patient characteristics. The data collection tools for all data elements relevant to the current study were identical and the facilities participating in phase 2 were the same as in phase 1, with the exception of eight facilities that dropped out between phases and one facility that was added.

### Definition of PD-related peritonitis

PD-related peritonitis was diagnosed in accordance with the criteria provided in the ISPD guideline [5], in having at least two of the following three findings: abdominal pain and/or cloudy dialysate effluent; white cell count in dialysis effluent >100/μl or >0.1 × 10^9^/l (after a dwell time ≥2 h), with >50% polymorphonuclear leukocytes; and positive culture results from the dialysis effluent. Exit site infection was also diagnosed using the criteria of the ISPD guideline [7] as the presence of purulent discharge with or without skin erythema at the catheter–epidermal interface.

Peritonitis cure was defined as the absence of a subsequent peritonitis event (relapse or recurrence, as defined previously at Al Sahlawi *et al.* [3]), PD catheter removal or death during the 50 days following the onset of a peritonitis episode, or the absence of permanent HD transfer (either a transfer to HD that was initially indicated as permanent or a transfer to HD that was initially indicated as temporary with no return to PD within 84 days).

### Variables

Patient demographics and comorbid conditions were captured at study enrolment. Routine laboratory findings, PD treatment-related data and summaries of peritonitis episodes were collected longitudinally throughout the study. Data were transcribed from medical records and entered into a web-based data collection tool. Two facility-level questionnaires were distributed annually to capture the practice patterns of the facility from the perspectives of both the medical director and the nurse study coordinator.

The outcome of interest was the time to first peritonitis episode caused by any organism. For each peritonitis episode, an infection worksheet capturing the date of first presentation and causative organism (or culture-negative case) was completed. Peritonitis episodes were also ascertained from facility-reported hospitalizations in which a diagnosis of peritonitis was indicated as a cause of hospitalization. Peritonitis episodes ascertained from hospitalization records alone were assumed to have the date of first presentation as the date of admission and unknown causative organisms. A peritonitis episode that occurred within 21 days of the initial episode was considered part of the same episode, as described previously [3].

### Statistical analysis

Crude facility peritonitis rates were estimated using the total count of reported peritonitis episodes in the facility divided by the total patient follow-up time in the facility. Descriptive statistics were used to summarize the demographic and clinical characteristics of patients as well as facility characteristics, both overall and by tertile of the facility peritonitis rate.

Cox proportional hazards survival models were used to investigate the associations of patient- and facility-level characteristics/practice patterns with the time to first peritonitis episode. Models were stratified by PDOPPS study phase, accounting for facility clustering using a robust sandwich covariance estimator. Time at risk for each patient started at PDOPPS enrolment and continued until the earliest of the following: first peritonitis episode during the study or at the end of patient follow-up [due to facility transfer, kidney transplantation, transfer to HD for >84 days (censored at date of transfer), withdrawal, death, loss to follow-up or administrative study end]. Models were sequentially adjusted for the prespecified exposures and potential confounders listed in the tables. Selected facility characteristics/practice patterns were explored as risk factors for peritonitis, first in individual unadjusted models, then after adjusting for patient case mix.

The proportion of missing data for variables of interest was <10%. Missing data for all independent variables in Cox models were multiply imputed by chained equations [8] and results from 20 imputed data sets were combined for the final analysis using Rubin's formula [9]. All statistical analyses were conducted using SAS version 9.4 (SAS Institute, Cary, NC, USA).

## RESULTS

### Patient characteristics

**Figure 1:**
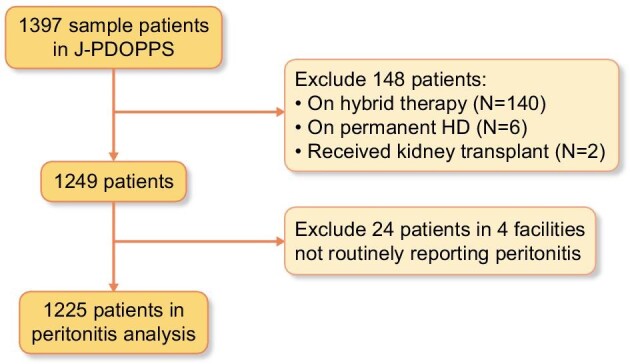
Consort diagram of the study cohort.

From among the 1397 Japan PDOPPS sample patients, patients were excluded from analysis for the following reasons: receiving hybrid (PD + HD) therapy (*n* = 140) at study enrolment, transfer to permanent HD (*n* = 6) or kidney transplant (*n* = 2) within the first 4 months of study enrolment. An additional 24 patients were excluded in facilities that did not routinely report peritonitis. A total of 1225 patients from 50 (28 unique) PD sites were included in the final analytic cohort (Fig. 1). The median age was 65 years [interquartile range (IQR) 55–73] and 33% were female.

The median duration of receiving PD was 1.0 year (IQR 0.05–2.88) and 83% of patients were able to produce a urine volume >200 ml/day. CAPD was the predominant PD modality (60% of patients) and icodextrin-based solution was used in 45% of patients.

#### Peritonitis rate

**Figure 2:**
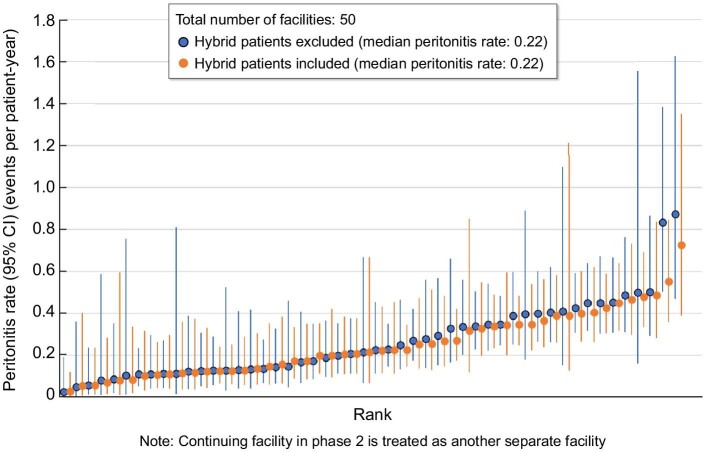
Distribution of facility peritonitis rates in Japan PDOPPS (2014–2022).

Over a median follow-up of 1.52 years (IQR 0.86–2.34), a total of 539 peritonitis episodes experienced by 364 patients were reported. The country crude peritonitis rate was 0.27 episodes/patient-year, with facility rates ranging from 0.03 to 0.87 episodes/patient-year (Fig. 2). A total of 44 episodes (8%) of peritonitis involved concomitant exit site infection and the frequency of this complication was lowest in those facilities with the lowest rates of peritonitis. The most frequent class of causative organism for all peritonitis episodes was Gram-positive bacteria (39%; Table 1). The percentage of peritonitis cases that were culture negative (20%) was higher than the 15% recommended in the ISPD guideline and tended to be lower in facilities with the lowest rates of peritonitis (Table 1). The overall peritonitis cure rate was 68%, ranging from 76% in facilities within the lowest tertile of peritonitis rates to 65% in facilities within the highest tertile of peritonitis rates.

**Table 1:** Proportion of episodes with concomitant exit site and organism class-specific infection, overall and by tertile of facility peritonitis rate.

		Facility peritonitis rate (episodes/patient-year) tertile		
Characteristics	Overall	1 (0.03–0.14)	2 (0.15–0.34)	3 (0.35–0.87)
Patients, *n*	1226	318	462	446
Peritonitis episodes, *n*	539	55	181	303
Episodes with concomitant exit site infection, %	8	4	10	8
Peritonitis organism class, %				
Gram positive	39	46	38	39
Gram negative	14	24	11	15
Culture negative	20	6	25	20
Polymicrobial	5	4	5	5
Yeast	0.4	1.8	0	0.3
Other	22	20	22	22
Particular organisms, %				
Coagulase negative *Staphylococcus*	5	4	6	5
*Staphylococcus aureus*	10	6	10	10
*Pseudomonas aeruginosa*	4	6	3	4
Peritonitis cure rate, %	68	76	70	65
The odds ratio (95% CI) of cure by tertile of facility peritonitis rate				
	Facility peritonitis rate (episodes/patient-year) tertile			
	1 (0.03–0.14)	2 (0.15–0.34)	3 (0.35–0.87)	
Model 1	1.75 (0.86–3.59)	1.19 (0.78–1.82)	1 (ref)	
Model 2	1.36 (0.64–2.91)	1.27 (0.80–2.02)	1 (ref)	
Model 3	1.36 (0.62–2.97)	1.29 (0.81–2.05)	1 (ref)	

Model 1: unadjusted,

Model 2: adjusted for age, gender, PD vintage, BMI and comorbidities.

Model 3: model 2 + PD modality and icodextrin use.

Patient characteristics by tertile of facility peritonitis rate are summarized in Table 2. Patients in facilities with higher peritonitis rates tended to be older with shorter PD vintage, have greater use of CAPD, have coronary artery disease or other cardiovascular diseases and have slightly worse nutritional indicators [e.g. lower body mass index (BMI), serum albumin and phosphorus]. Overall usage rates of proton pump inhibitors (PPIs) and histamine-2 receptor blockers (H2RBs) were 41% and 4%, respectively. Higher facility peritonitis rates tended to be associated with a higher frequency of hypokalaemia.

**Table 2:** Patient characteristics in the Japan PDOPPS, overall and by tertile of facility peritonitis rate.

Patient/treatment		Facility peritonitis rate (episodes/patient-year) tertile		
characteristics	Overall	1 (0.03–0.14)	2 (0.15–0.34)	3 (0.35–0.87)
Patients, *n*	1226	318	462	446
Demographics				
Age (years), median (IQR)	65 (55–73)	63 (53–71)	65 (56–73)	67 (57–75)
Female, %	33	33	33	33
Time on PD (years), median (IQR)	1.0 (0.0–2.9)	1.6 (0.1–3.5)	0.9 (0.1–2.7)	0.8 (0.0–2.7)
Urine volume >200 ml/day, %	83	83	84	82
BMI (kg/m^2^), mean (SD)	23.1 (3.6)	23.2 (4.0)	23.1 (3.4)	22.9 (3.5)
Current smoker, %	11	10	11	12
Comorbidities, %				
Diabetes	40	32	45	41
Hypertension	95	95	95	94
Congestive heart failure	18	14	25	13
Coronary artery disease	15	10	15	19
Cerebrovascular disease	13	12	14	13
Peripheral vascular disease	7	6	7	7
Other cardiovascular disease	13	7	17	14
PD prescription, %				
CAPD (versus APD)	60	41	62	70
Icodextrin	45	46	44	46
Labs				
Albumin (g/dl), mean (SD)	3.3 (0.5)	3.3 (0.5)	3.4 (0.5)	3.2 (0.5)
Haemoglobin (g/dl), mean (SD)	11.0 (1.3)	11.0 (1.2)	11.0 (1.3)	10.9 (1.4)
Phosphorus (mg/dl), mean (SD)	5.0 (1.2)	5.2 (1.3)	5.0 (1.2)	4.9 (1.2)
Potassium (mEq/l), mean (SD)	4.2 (0.7)	4.2 (0.7)	4.3 (0.6)	4.2 (0.8)
Hypokalaemia^a^, %	13	13	7	18
Medications, %				
PPI	41	40	44	38
H2RB	4	2	4	4

a Defined as a serum potassium level <3.5 mEq/l.

Table 3 presents facility characteristics for the Japan PDOPPS. The median facility size was 31 patients. Facilities with a higher peritonitis rate tended to have a larger patient:physician ratio (>10), late start of PD education/training after PD catheter insertion, lower likelihood of providing antibiotic prophylaxis (especially for complicated dental procedures, gynaecological procedures and lower endoscopy) and more frequent regular medical visits. No relationship was evident between facility peritonitis rates and gastric acid suppression (using PPIs or H2RBs).

**Table 3:** Facility characteristics in the Japan PDOPPS, overall and by tertile of facility peritonitis rate.

		Facility peritonitis rate (episodes/patient-year) tertile		
Facility characteristic/practice	Overall	1 (0.03–0.14)	2 (0.15–0.34)	3 (0.35–0.87)
Facilities, *n*	50	17	17	16
Facility size, median (IQR)	31 (23–41)	30 (21–41)	32 (29–40)	29 (26–43)
Patient:physician ratio, %				
<4	22	33	31	0
4–6	20	17	25	15
7–9	32	42	31	23
≥10	27	8	13	62
Patient:nurse ratio, %				
<4	34	33	31	38
4–6	17	17	19	15
7–9	37	50	25	38
≥10	12	0	25	8
PD education				
Total time (hours), median (IQR)	5 (3–10)	3 (3–5)	9 (3–10)	8 (5–10)
Training format, %				
One-on-one	90	100	88	85
Group	0	0	0	0
Combination	10	0	13	15
Timing, %				
Prior to PD catheter insertion	59	83	56	38
1 week after PD catheter insertion	27	17	31	31
≥2 weeks after PD catheter insertion	2	0	6	0
Other	12	0	6	31
Location of initial training, %				
Home	0	0	0	0
Facility	97	100	94	100
Combination	3	0	6	0
Antibiotic prophylaxis practice^a^, %				
At the time of PD catheter insertion	90	100	88	75
Routine dental procedures (i.e. cleaning)	3	0	6	0
Complicated dental procedures (i.e. tooth extraction)	73	87	71	50
Gynaecological procedures (i.e. hysteroscopy, endometrial ablation), %	48	53	47	38
Lower endoscopy (i.e. colonoscopy), %	58	80	53	25
Screening for *Staphylococcus aureus* nasal carriage, %				
Yes	23	7	35	25
No	78	93	65	75
Regular medical visits,^b^ %				
Once a week	2	0	0	8
Once, every 2–3 weeks	34	25	38	38
Once a month	63	75	63	54
PD/other prescription, median (IQR)				
Facility % icodextrin	54 (36–70)	55 (40–67)	50 (36–60)	60 (34–76)
Facility % PPIs	42 (32–47)	38 (25–53)	43 (38–46)	34 (23–56)
Facility % H2RBs	0 (0–6)	0 (0–0)	2 (0–6)	2 (0–7)

a Procedures/situations where antibiotic prophylaxis is used or recommended for PD patients at your centre; Japan PDOPPS medical director survey.

b Regular medical visits is not at the patient level.

### Associations between patient-level factors and outcome

Adjusted associations between patient-level factors and time to first episode of any peritonitis are shown in Table 4. Age {hazard ratio [HR] 1.07 per 5-year increase [95% confidence interval (CI) 1.01–1.14]} and CAPD use [HR 1.31 versus APD use (95% CI 1.05–1.63)] were positively associated with experiencing any peritonitis, whereas the hazard of experiencing any peritonitis was 37% lower for patients with a higher serum albumin level [HR 0.63 per 1 g/dl higher (95% CI 0.48–0.82)]. Associations with other patient factors were not significant.

**Table 4:** Patient characteristics associated with time to first peritonitis; effect of progressive adjustment.

	HR (95% CI)				
Patient/treatment characteristics	Model 1	Model 2	Model 3	Model 4	Model 5
Demographics					
Age, per 5 years	1.11 (1.05–1.17)	1.10 (1.04–1.16)	1.07 (1.01–1.14)	1.07 (1.01–1.14)	1.07 (1.01–1.14)
Female	0.85 (0.67–1.09)	0.87 (0.67–1.13)	0.83 (0.63–1.09)	0.83 (0.63–1.08)	0.82 (0.63–1.08)
Time on PD (per year)	0.98 (0.95–1.02)	0.98 (0.94–1.03)	1.00 (0.96–1.04)	1.00 (0.96–1.05)	1.00 (0.96–1.05)
BMI (per kg/m^2^)	1.01 (0.97–1.04)	1.00 (0.97–1.04)	1.02 (0.98–1.06)	1.02 (0.98–1.06)	1.02 (0.98–1.06)
Current smoker		1.13 (0.86–1.51)	1.17 (0.86–1.59)	1.15 (0.84–1.57)	1.15 (0.84–1.56)
Comorbidities					
Diabetes		0.98 (0.77–1.27)	0.93 (0.72–1.20)	0.93 (0.72–1.20)	0.93 (0.72–1.20)
Hypertension		1.13 (0.72–1.76)	1.18 (0.77–1.80)	1.14 (0.74–1.74)	1.14 (0.74–1.73)
Congestive heart failure		0.98 (0.73–1.32)	1.01 (0.73–1.38)	1.02 (0.74–1.40)	1.01 (0.74–1.39)
Coronary artery disease		1.04 (0.75–1.45)	1.03 (0.72–1.47)	0.98 (0.67–1.41)	0.97 (0.67–1.41)
Cerebrovascular disease		0.85 (0.63–1.15)	0.79 (0.57–1.09)	0.78 (0.56–1.08)	0.78 (0.57–1.08)
Peripheral vascular disease		1.10 (0.71–1.69)	1.13 (0.76–1.68)	1.12 (0.75–1.68)	1.12 (0.75–1.67)
Other cardiovascular disease		1.31 (0.93–1.83)	1.18 (0.87–1.60)	1.19 (0.89–1.61)	1.19 (0.89–1.61)
PD prescription					
CAPD (versus APD)			1.30 (1.04–1.63)	1.31 (1.05–1.63)	1.31 (1.05–1.62)
Icodextrin			0.90 (0.72–1.14)	0.90 (0.71–1.13)	0.90 (0.71–1.13)
Labs					
Albumin (per g/dl)			0.63 (0.48–0.83)	0.63 (0.48–0.82)	0.63 (0.48–0.82)
Haemoglobin (per g/dl)			0.98 (0.89–1.08)	0.98 (0.89–1.08)	0.98 (0.89–1.08)
Phosphorus (per mg/dl)			0.92 (0.83–1.02)	0.93 (0.84–1.03)	0.93 (0.84–1.03)
Potassium (mEq/l)					
<3.5			1.11 (0.80–1.54)	1.11 (0.80–1.53)	1.10 (0.80–1.53)
3.5–5			1 (ref)	1 (ref)	1 (ref)
>5			1.40 (0.97–2.04)	1.37 (0.93–2.02)	1.36 (0.92–2.01)
Medications					
PPI				1.12 (0.90–1.41)	
H2RB				1.29 (0.54–3.08)	
Any gastric acid suppressant					1.14 (0.90–1.46)

Number of patients: 1225; number of events: 364.

Model 1: individual characteristics only.

Model 2: model 1 + current smoking status and seven comorbidities.

Model 3: model 2 + PD prescription (i.e. PD type, icodextrin use) and labs (i.e. albumin, haemoglobin, phosphorus and potassium).

Model 4: model 3 + medications (i.e. PPI and H2RB) use.

Model 5: model 3 + any gastric acid suppressant use.

### Associations between facility-level factors and outcome

Table 5 presents the associations of facility-level factors with time to first episode of any peritonitis. A lower risk of experiencing any peritonitis was observed with adoption of antibiotic prophylaxis practice in a facility, particularly at the time of PD catheter insertion [HR 0.63 (95% CI 0.51–0.78)], complicated dental procedures [HR 0.74 (95% CI 0.57–0.95)] and lower endoscopy [HR 0.69 (95% CI 0.54–0.89)]. A higher risk was observed with more frequent regular medical visits. Associations with other facility factors including patient:nurse ratio, PD training/education time, screening for *Staphylococcus aureus* nasal carriage and prescription of icodextrin or PPIs were not significant.

There was no significant difference in the results after excluding two sites with severe outlier peritonitis rates (>0.5 in Fig. 2) from the samples (Supplementary Information 1).

**Table 5:** Facility characteristics/practices associated with patient time to first peritonitis.

	HR (95% CI)	
Facility characteristic/practice	Unadjusted	Case mix adjusted^a^
Patients and staffing		
Facility size		
Small (<23)	0.93 (0.64–1.36)	0.91 (0.64–1.29)
Medium (23–41)	1 (ref)	1 (ref)
Large (>41)	1.20 (0.80–1.79)	1.17 (0.82–1.68)
Patient:physician ratio		
<4	0.67 (0.41–1.09)	0.70 (0.46–1.07)
4–6	1 (ref)	1 (ref)
7–9	0.89 (0.56–1.44)	0.95 (0.64–1.41)
≥10	1.32 (0.82–2.12)	1.34 (0.89–2.02)
Patient:nurse ratio		
<4	1.15 (0.78–1.70)	1.06 (0.71–1.59)
4–6	1 (ref)	1 (ref)
7–9	1.13 (0.73–1.74)	1.10 (0.72–1.66)
≥10	1.33 (0.68–2.59)	1.23 (0.61–2.47)
PD education		
Total time (hours)		
3–10	1 (ref)	1 (ref)
>10	1.02 (0.70–1.50)	1.08 (0.70–1.65)
Timing		
Prior to PD catheter insertion	0.81 (0.57–1.16)	0.84 (0.61–1.15)
1 or 2 weeks after PD catheter insertion	1 (ref)	1 (ref)
Other	1.19 (0.93–1.54)	1.17 (0.90–1.54)
Antibiotic prophylaxis practice (yes versus no)		
At the time of PD catheter insertion	0.62 (0.49–0.77)	0.63 (0.51–0.78)
Complicated dental procedures (i.e. tooth extraction)	0.73 (0.54–0.99)	0.74 (0.57–0.95)
Gynaecological procedures^b^ (i.e. hysteroscopy, endometrial ablation)	1.00 (0.62–1.63)	0.98 (0.61–1.57)
Lower endoscopy (i.e. colonoscopy)	0.66 (0.51–0.87)	0.69 (0.54–0.89)
Screening for *Staphylococcus aureus* nasal carriage		
Yes versus no	1.14 (0.82–1.59)	1.03 (0.78–1.37)
Regular medical visits^c^		
Once a month versus more often than once a month	0.73 (0.54–0.99)	0.73 (0.57–0.95)
PD/other prescription		
Facility % icodextrin (per %)	0.94 (0.37–2.39)	0.95 (0.40–2.24)
Facility % PPIs (per %)	1.00 (0.29–3.48)	1.14 (0.40–3.24)

Number of patients: 1225; number of events: 364.

a Adjusted for patient age, sex, time on PD, diabetes, congestive heart failure and CAPD.

b Restricted to female patients

c Regular medical visits is not at the patient level.

## DISCUSSION

PD-related peritonitis is a major cause of withdrawal from PD and an important cause of death [1, 3, 10, 11]. From the perspective of improving outcomes for PD patients, the identification and mitigation of risk factors for PD-related peritonitis is important. International differences in risk factors for PD-related peritonitis have been reported [1, 2]. One notable finding is that the incidence of fungal peritonitis was lowest in Japan among countries in the PDOPPS [1], where prophylaxis against fungal peritonitis is not generally performed [2] despite being recommended by the ISPD guideline [5]. This may be due to ethnic, cultural or practical differences. Many risk factors have been reported but frequently seem dependent on the countries and cohorts. The present study investigated characteristic risk factors for PD-related peritonitis in Japan to facilitate the development of countermeasures and clarify the meaning of comprehensive risk evaluation in Japan.

Importantly, facility peritonitis rates showed a wide distribution, from 0.03 to 0.87 episodes per patient-year in Japan (Fig. 2), suggesting that the quality of practices and preventive measures against PD differ markedly among Japanese facilities. Similar phenomena have been observed in many countries and variations in outcomes still exist across PD facilities [12]. We hypothesized that such differences are attributable to both institutional (centre-level) and individual (patient-level) characteristics. We therefore investigated both centre- and patient-level characteristics associated with time to first peritonitis. For the former, we divided facility peritonitis rates into tertiles.

Several modifiable risk factors were identified from centre-level characteristics. Antibiotic prophylaxis is recommended just before PD catheter insertion, routine and complicated dental procedures, gynaecological procedures and colonoscopy with or without polypectomy [5]. Such prophylactic practices are reportedly conducted at rates of 89%, 7%, 68%, 32% and 36%, respectively, in Japan [2], but whether these data are reflected in the occurrence of peritonitis has been unclear. The present study indicated that antibiotic prophylaxis at the time of PD catheter insertion (HR 0.63), complicated dental procedures (HR 0.74) and lower endoscopy (HR 0.69) effectively prevent peritonitis in Japan (Table 4). Antibiotic prophylaxis before these procedures as a facility practice was clearly associated with reduced rates of peritonitis. A strong association between recent exit site infection and development of peritonitis has been reported [13, 14]. In the present cohort, concomitant exit site infection tended to be low in the lowest tertile of facility peritonitis rates (Table 1). This suggests that exit site infection is a possible risk factor for peritonitis in a Japanese PDOPPS cohort in which 96% were not on antimicrobial ointment application therapy [2]. It is interesting to note that the PD centres with higher peritonitis rates tended to have both a higher proportion of culture-negative peritonitis and lower rates of cure following peritonitis (Table 1). We speculate that one explanation for these findings is that the centres with lower peritonitis rates in general may have had more optimal outcomes in part because of greater adherence to a number of ISPD prevention and treatment recommendations and perhaps more quality improvement practices in place that resulted in a number of improved outcomes. For example, having a small percentage of culture-negative cases allows for an effective antibiotic to be prescribed for a larger fraction of peritonitis episodes, resulting in a greater likelihood of having higher peritonitis cure rates compared with centres having a high percentage of culture-negative peritonitis cases.

Whether CAPD represents a risk factor for PD-related peritonitis has remained controversial [15, 16]. The PDOPPS, which included a Japanese cohort, indicated CAPD is a peritonitis risk factor [1], but a systematic review showed it was not [17, 18]. In Japan, the use of APD versus CAPD was lower compared with other PDOPPS countries except Thailand [19]. Selection of PD in Japan is based on shared decision-making and the higher prevalence of CAPD may be related to the preference for incremental PD. CAPD was a risk factor in the present cohort (Table 4) and thus should be given attention as a risk factor in Japan.

Programs training patients in PD are considered a centre-level characteristic, but may represent the most difficult area for which to establish evidence of effectiveness. In recent PDOPPS analyses, evidence supporting an association between PD training practices and peritonitis remained lacking [4]. Factors for the optimal training program have yet to be determined, including those of when, where, how or for how long training should be conducted [4]. In the seven countries that participated in the PDOPPS, marked variations have been seen in training practices, education systems and the roles of nurses. We therefore hypothesized that an ‘optimal’ program for PD training may be more easily established using analyses from a single country. Although facilities with lower peritonitis rates tend to provide PD education prior to PD catheter insertion, the early PD education did not have a significant effect on avoiding the occurrence of peritonitis. Unfortunately, no training factors, including timing of PD education or duration of PD education, were clearly associated with peritonitis risk (Tables 3 and 5). Proposing options for an optimal training program thus remains difficult. Surprisingly, more frequent regular medical visits, which allow for more intensive assessment and education time, were associated with higher peritonitis rates (Table 5). The characteristics of facilities typically having visits more than once a month tended to also have a higher patient:physician ratio (Supplementary Information 2), which may lead to a lack of careful examination and education for patients. However, the PDOPPS did not collect further data on this, thus it was difficult to determine a clear reason. This suggests that training and quality of practice may be more important than the number of medical visits. In terms of training, the content of the training curriculum and the teaching skills of the trainers may need to be included in evaluations for the establishment of PD training programs. Recently, the importance of retraining for the long-term has been emphasized [20–22]. Consideration of these factors is likely to prove important in establishing effective PD training programs.

Previously reported modifiable patient-level characteristics include gastrointestinal conditions such as hypokalaemia [23–26], constipation and the use of gastric acid suppressants [27] like PPIs [28] and H2RBs [27], which are commonly prescribed worldwide. Hypokalaemia has been reported as a risk factor for PD-related peritonitis in several articles, including a report on the PDOPPS [23]. Many possible mechanisms have been postulated, including gastrointestinal dysmotility, intestinal bacterial overgrowth and translocation of bacteria from the intestine to the peritoneal cavity [24]. Fortunately, the incidence of hypokalaemia is low in Japan [23] and this was not a risk factor in the present study (Table 4). Usage rates of H2RBs are also low in Japan. Neither PPIs nor H2RBs were risk factors for peritonitis in the present Japanese cohort (Table 4).

Many comorbidities and patient-level characteristics were assessed as potential risk factors for PD-related peritonitis. Several studies have reported diabetes as an independent risk factor for PD-related peritonitis [16, 29–33], but diabetes was not a risk factor in the present cohort (Table 4). The cause was considered to be bacterial overgrowth due to decreased intestinal motility, a decrease of peritoneal defence mechanisms due to interference with the migration of phagocytic cells into the peritoneum and phagocytic activity [34]. Other comorbidities including heart failure [35–37], cardiovascular diseases [30, 38, 39], cerebrovascular diseases [30, 32, 35], hypertension [35], high BMI [40–43], smoking [31] and low haemoglobin [30] have been reported but were not important risk factors in the present Japanese cohort (Table 4). Risk factors clearly differ among countries and cohorts. Identifying the specific risk factors within a given country may prove useful for establishing country-specific countermeasures, developing future versions of PD guidelines [44, 45] and improving clinical practices nationwide.

The strengths of the present study were the prospective design and the use of a multicentre cohort of patients from a single country, Japan. However, some limitations must be acknowledged. First, the number of variables in the PDOPPS database was limited, so the possibility of unmeasured confounders cannot be excluded. Second, some biases may have been present in the centre selection criteria. Third, the observational design only allowed identification of associations. Fourth, some data were missing.

In conclusion, potentially modifiable risk factors for PD-related peritonitis can differ among countries. Clarification of characteristic country-level risk factors for PD-related peritonitis from a comprehensive perspective is likely to provide useful insights for developing countermeasures against peritonitis and future versions of guidelines. Based on these findings, further research is needed, particularly on training and quality of practice.

## Supplementary Material

sfae202_Supplemental_File

## Data Availability

The data underlying this article will be shared upon reasonable request to the corresponding author.

## APPENDIX

Japan PDOPPS study committee: Hideki Kawanishi (Kidney Disease and Blood Purification Therapy, Akane-Foundation, Tsuchiya General Hospital, Hiroshima, Japan), Jun Minakuchi (Department of Kidney Disease, Kawashima Hospital, Tokushima, Japan), Tadashi Tomo (Clinical Engineering Research Center, Oita University, Oita, Japan), Ken Tsuchiya (Department of Blood Purification, Tokyo Women's Medical University, Tokyo, Japan), Kousaku Nitta (Department of Medicine, Kidney Center, Tokyo Women's Medical University, Tokyo, Japan), Munekazu Ryuzaki (Division of Nephrology, Department of Internal Medicine, Tokyo Saiseikai Central Hospital, Tokyo, Japan), Mizuya Fukazawa (Department of Urology, Faculty of Medicine, University of Yamanashi, Chuo, Japan), Yasuhiro Ito (Department of Nephrology and Rheumatology, Aichi Medical University, Nagakute, Japan), Hidetomo Nakamoto (Department of General Internal Medicine, Saitama Medical University, Saitama, Japan) and Akihiro Yamashita (Department of Chemical Science and Technology, Faculty of Bioscience and Applied Chemistry, Hosei University, Tokyo, Japan).

## References

[1] Perl J, Fuller DS, Bieber BA et al. Peritoneal dialysis-related infection rates and outcomes: results from the Peritoneal Dialysis Outcomes and Practice Patterns Study (PDOPPS). Am J Kidney Dis 2020;76:42–53. 10.1053/j.ajkd.2019.09.016 31932094

[2] Boudville N, Johnson DW, Zhao J et al. Regional variation in the treatment and prevention of peritoneal dialysis-related infections in the Peritoneal Dialysis Outcomes and Practice Patterns Study. Nephrol Dial Transplant 2019;34:2118–26. 10.1093/ndt/gfy204 30053214 PMC6887924

[3] Al Sahlawi M, Zhao J, McCullough K et al. Variation in peritoneal dialysis–related peritonitis outcomes in the Peritoneal Dialysis Outcomes and Practice Patterns Study (PDOPPS). Am J Kidney Dis 2022;79:45–55.e1. 10.1053/j.ajkd.2021.03.022 34052357

[4] Cheetham MS, Zhao J, McCullough K et al. International peritoneal dialysis training practices and the risk of peritonitis. Nephrol Dial Transplant 2022;37:937–49. 10.1093/ndt/gfab298 34634100

[5] Li PK, Chow KM, Cho Y et al. ISPD peritonitis guideline recommendations: 2022 update on prevention and treatment. Perit Dial Int 2022;42:110–53. 10.1177/08968608221080586 35264029

[6] Perl J, Davies SJ, Lambie M et al. The Peritoneal Dialysis Outcomes and Practice Patterns Study (PDOPPS): unifying efforts to inform practice and improve global outcomes in peritoneal dialysis. Perit Dial Int 2016;36:297–307. 10.3747/pdi.2014.00288 26526049 PMC4881793

[7] Szeto CC, Li PK, Johnson DW et al. ISPD catheter-related infection recommendations: 2017 update. Perit Dial Int 2017;37:141–54. 10.3747/pdi.2016.00120 28360365

[8] Raghunathan T, Lepkowski J, Hoewyk J et al. A multivariate technique for multiply imputing missing values using a sequence of regression models. Surv Methodol 2000;27:85–95.

[9] Toutenburg H, Rubin DB. Multiple imputation for nonresponse in surveys. Stat Pap (Berl) 1990;31:180. 10.1007/BF02924688

[10] Mizuno M, Ito Y, Tanaka A et al. Peritonitis is still an important factor for withdrawal from peritoneal dialysis therapy in the Tokai area of Japan. Clin Exp Nephrol 2011;15:727–37. 10.1007/s10157-011-0471-8 21691738

[11] Mizuno M, Ito Y, Suzuki Y et al. Recent analysis of status and outcomes of peritoneal dialysis in the Tokai area of Japan: the second report of the Tokai peritoneal dialysis registry. Clin Exp Nephrol 2016;20:960–71. 10.1007/s10157-016-1249-9 26951303

[12] Perl J, Fuller DS, Boudville N et al. Optimizing peritoneal dialysis-associated peritonitis prevention in the United States: from standardized peritoneal dialysis-associated peritonitis reporting and beyond. Clin J Am Soc Nephrol 2020;16:154–61. 10.2215/CJN.11280919 32764025 PMC7792655

[13] Lloyd A, Tangri N, Shafer LA et al. The risk of peritonitis after an exit site infection: a time-matched, case-control study. Nephrol Dial Transplant 2013;28:1915–21. 10.1093/ndt/gft002 23382265

[14] van Diepen AT, Tomlinson GA, Jassal SV. The association between exit site infection and subsequent peritonitis among peritoneal dialysis patients. Clin J Am Soc Nephrol 2012;7:1266–71. 10.2215/CJN.00980112 22745277 PMC3408122

[15] Akman S, Bakkaloglu SA, Ekim M et al. Peritonitis rates and common microorganisms in continuous ambulatory peritoneal dialysis and automated peritoneal dialysis. Pediatr Int 2009;51:246–9. 10.1111/j.1442-200X.2008.02693.x 19405925

[16] Duquennoy S, Béchade C, Verger C et al. Is peritonitis risk increased in elderly patients on peritoneal dialysis? Report from the French Language Peritoneal Dialysis Registry (RDPLF). Perit Dial Int 2016;36:291–6. 10.3747/pdi.2014.00154 26634564 PMC4881792

[17] Rabindranath KS, Adams J, Ali TZ et al. Continuous ambulatory peritoneal dialysis versus automated peritoneal dialysis for end-stage renal disease. Cochrane Database Syst Rev 2007;2007:CD006515. 17443624 10.1002/14651858.CD006515 PMC6669246

[18] Lan PG, Johnson DW, McDonald SP et al. The association between peritoneal dialysis modality and peritonitis. Clin J Am Soc Nephrol 2014;9:1091–7. 10.2215/CJN.09730913 24626434 PMC4046732

[19] Wang AYM, Zhao J, Bieber B et al. International comparison of peritoneal dialysis prescriptions from the Peritoneal Dialysis Outcomes and Practice Patterns Study (PDOPPS). Perit Dial Int 2020;40:310–9. 10.1177/0896860819895356 32063209

[20] Chang JH, Oh J, Park SK et al. Frequent patient retraining at home reduces the risks of peritoneal dialysis-related infections: a randomised study. Sci Rep 2018;8:12919. 10.1038/s41598-018-30785-z 30150627 PMC6110747

[21] Xu Y, Zhang Y, Yang B et al. Prevention of peritoneal dialysis-related peritonitis by regular patient retraining via technique inspection or oral education: a randomized controlled trial. Nephrol Dial Transplant 2020;35:676–86. 10.1093/ndt/gfz238 31821491

[22] Ljungman S, Jensen JE, Paulsen D et al. Retraining for prevention of peritonitis in peritoneal dialysis patients: a randomized controlled trial. Perit Dial Int 2020;40:141–52. 10.1177/0896860819887626 32063220

[23] Davies SJ, Zhao J, Morgenstern H et al. Low serum potassium levels and clinical outcomes in peritoneal dialysis—international results from PDOPPS. Kidney Int Rep 2021;6:313–24. 10.1016/j.ekir.2020.11.021 33615056 PMC7879114

[24] Nakai K, Saito K, Fujii H et al. Impact of hypokalemia on peritonitis in peritoneal dialysis patients: a systematic review. Renal Replace Ther 2017;3:43. 10.1186/s41100-017-0128-5

[25] Ribeiro SC, Figueiredo AE, Barretti P et al. Low serum potassium levels increase the infectious-caused mortality in peritoneal dialysis patients: a propensity-matched score study. PLoS One 2015;10:e0127453. 10.1371/journal.pone.0127453 26091005 PMC4474697

[26] Liu D, Lin Y, Gong N et al. Degree and duration of hypokalemia associated with peritonitis in patients undergoing peritoneal dialysis. Int J Clin Pract 2021;75:e14188. 10.1111/ijcp.14188 33783932

[27] Zhong HJ, Lin D, Lu ZY et al. Use of gastric-acid suppressants may be a risk factor for enteric peritonitis in patients undergoing peritoneal dialysis: a meta-analysis. J Clin Pharm Ther 2019;44:209–15. 10.1111/jcpt.12769 30332507

[28] Maeda S, Yamaguchi M, Maeda K et al. Proton pump inhibitor use increases the risk of peritonitis in peritoneal dialysis patients. PLoS One 2019;14:e0224859. 10.1371/journal.pone.0224859 31697753 PMC6837385

[29] Nishina M, Yanagi H, Kakuta T et al. A 10-year retrospective cohort study on the risk factors for peritoneal dialysis-related peritonitis: a single-center study at Tokai University Hospital. Clin Exp Nephrol 2014;18:649–54. 10.1007/s10157-013-0872-y 24085653

[30] Zhang R, Zhang X, Tang X et al. The association between diabetes coexisting with low levels of high-density lipoprotein cholesterol and peritoneal dialysis-related peritonitis. Diabetol Metab Syndr 2022;14:60. 10.1186/s13098-022-00832-x 35488249 PMC9052536

[31] Terada K, Sumi Y, Aratani S et al. Smoking is a risk factor for endogenous peritonitis in patients undergoing peritoneal dialysis. J Nippon Med Sch 2021;88:461–6. 10.1272/jnms.JNMS.2021_88-604 33692295

[32] Chen HL, Tarng DC, Huang LH. Risk factors associated with outcomes of peritoneal dialysis in Taiwan: an analysis using a competing risk model. Medicine (Baltimore) 2019;98:e14385. 10.1097/MD.0000000000014385 30732176 PMC6380716

[33] Chaudhry RI, Chopra T, Fissell R et al. Strategies to prevent peritonitis after procedures: our opinions. Perit Dial Int 2019;39:315–9. 10.3747/pdi.2018.00148 31296777

[34] Hodgson K, Morris J, Bridson T et al. Immunological mechanisms contributing to the double burden of diabetes and intracellular bacterial infections. Immunology 2015;144:171–85. 10.1111/imm.12394 25262977 PMC4298412

[35] Chung MC, Yu TM, Wu MJ et al. Impact of peritoneal dialysis-related peritonitis on PD discontinuation and mortality: a population-based national cohort study. Perit Dial Int 2022;42:194–203. 10.1177/08968608211018949 34100316

[36] Santhakumaran T, Samad N, Fan SL. Hydration status measured by BCM: a potential modifiable risk factor for peritonitis in patients on peritoneal dialysis. Nephrology (Carlton) 2016;21:404–9. 10.1111/nep.12622 26369571

[37] Carvalho Fiel D, Pérez-Fontán M, López Iglesias A et al. Persistent overhydration is associated with a significant risk of peritoneal infection by enteric pathogens in patients treated with peritoneal dialysis. Nefrologia (Engl Ed) 2019;39:638–45. 10.1016/j.nefroe.2019.01.011 31023497

[38] Banno T, Shima H, Kawahara K et al. Risk factors for peritoneal dialysis withdrawal due to peritoneal dialysis-related peritonitis. Nephrol Ther 2021;17:108–13. 10.1016/j.nephro.2020.10.007 33495136

[39] Aldriwesh M, Alajroush L, Alangari R et al. A retrospective analysis of peritoneal dialysis-associated peritonitis at a single-care children's hospital in Saudi Arabia. Saudi J Kidney Dis Transpl 2021;32:735–43. 10.4103/1319-2442.336769 35102916

[40] Obi Y, Streja E, Mehrotra R et al. Impact of obesity on modality longevity, residual kidney function, peritonitis, and survival among incident peritoneal dialysis patients. Am J Kidney Dis 2018;71:802–13. 10.1053/j.ajkd.2017.09.010 29223620 PMC5970950

[41] Ong LM, Ch'ng CC, Wee HC et al. Risk of peritoneal dialysis-related peritonitis in a multi-racial Asian population. Perit Dial Int 2017;37:35–43. 10.3747/pdi.2015.00141 27147287

[42] McDonald SP, Collins JF, Rumpsfeld M et al. Obesity is a risk factor for peritonitis in the Australian and New Zealand peritoneal dialysis patient populations. Perit Dial Int 2004;24:340–6. 10.1177/089686080402400408 15335147

[43] Sato T, Anan G, Hirose T et al. The impact of preoperative risk factors on peritoneal dialysis-related peritonitis: a single-center prospective study in Japan. Medicina (Kaunas) 2022;58:313. 35208636 10.3390/medicina58020313 PMC8878486

[44] Ito Y, Ryuzaki M, Sugiyama H et al. Peritoneal dialysis guidelines 2019 part 1 (position paper of the Japanese Society for Dialysis Therapy). Renal Replace Ther 2021;7:40. 10.1186/s41100-021-00348-6

[45] Ryuzaki M, Ito Y, Nakamoto H et al. Peritoneal dialysis guidelines 2019 part 2: main text (position paper of the Japanese Society for Dialysis Therapy). Renal Replace Ther 2021;7:46. 10.1186/s41100-021-00361-9

