# Supplementary material for: Risk factors of peritoneal dialysis–related peritonitis in the Japan Peritoneal Dialysis Outcomes and Practice Patterns Study (PDOPPS)
Source: Clin Kidney J. 2024 Jul 1;17(7):sfae202. doi: 10.1093/ckj/sfae202 (PMC11267232; doi:10.1093/ckj/sfae202)
Supplement: sfae202_Supplemental_File [file sfae202_supplemental_file.docx]

**Supplementary Information**

# **Risk factors of peritoneal dialysis-related peritonitis in the Japan Peritoneal Dialysis Outcomes and Practice Patterns Study (PDOPPS)**

# **Authors**

# Yasuhiko Ito^1^, Charlotte Tu^2^, Makoto Yamaguchi^1^, Shigehisa Koide^3^, Munekazu Ryuzaki^4^, Brian Bieber^2^, Ronald L Pisoni^2^, Jeffrey Perl^5^, Jun Minakuchi^6^, Hideki Kawanishi^7,8^, and the Japan PDOPPS Study Committee

**Supplementary Information 1**.

**Supplementary Tables 1-5 were results after excluding 2 sites with severe outlier peritonitis rates from the analysis.**

**Supplementary Table 1.** Proportion of episodes with concomitant exit site and organism class-specific infection, overall and by tertile of facility peritonitis rate

**Supplementary Table 2.** Patient characteristics in Japan PDOPPS, overall and by tertile of facility peritonitis rate

**Supplementary Table 3.** Facility characteristics in Japan PDOPPS, overall and by tertile of facility peritonitis rate

**Supplementary Table 4.** Patient characteristics associated with time to first peritonitis: effect of progressive adjustment

**Supplementary Table 5.** Facility characteristics/practices associated with patient time to first peritonitis

**Supplementary Information 2**.

Facility characteristics by frequency of regular medical visits

**Supplementary Information 1**.

**Supplementary Table 1-5 were results after excluding 2 sites with severe outlier peritonitis rates from the analysis.**

**Supplementary Table 1.** Proportion of episodes with concomitant exit site and organism class-specific infection, overall and by tertile of facility peritonitis rate

|  | **Overall** | **Facility peritonitis rate (episodes/patient-year) tertile** | | |
| --- | --- | --- | --- | --- |
|  |  | **1 (0.03-0.13)** | **2 (0.14-0.34)** | **3 (0.34-0.50)** |
| Patients, n | 1200 | 273 | 469 | 458 |
| Number of peritonitis episodes | 514 | 47 | 164 | 303 |
| Episodes with concomitant exit site infection | 7% | 4% | 6% | 8% |
| Peritonitis organism class |  |  |  |  |
| Gram positive | 39% | 49% | 38% | 38% |
| Gram negative | 15% | 26% | 11% | 15% |
| Culture negative | 21% | 4% | 24% | 22% |
| Polymicrobial | 5% | 4% | 5% | 5% |
| Yeast | 0% | 2.1% | 0% | 0.3% |
| Other | 20% | 15% | 22% | 21% |
| Particular Organisms  Coagulase negative Staphylococcus |  |  |  |  |
|  | 5% | 2% | 6% | 6% |
| Staphylococcus Aureus | 9% | 6% | 11% | 8% |
| Pseudomonas aeruginosa | 4% | 6% | 4% | 4% |
| Peritonitis cure rate | 68% | 81% | 74% | 62% |

The odds ratio (95% CI) of cure by tertile of facility peritonitis rate

|  | Facility Peritonitis rate (episodes/patient-year) tertile | | |
| --- | --- | --- | --- |
|  | 1 (0.03-0.13) | 2 (0.14-0.34) | 3 (0.34-0.50) |
| Model 1 | 2.78 (1.19, 6.52) | 1.64 (1.05, 2.57) | 1 (ref) |
| Model 2 | 2.15 (0.87, 5.27) | 1.60 (0.99, 2.61) | 1 (ref) |
| Model 3 | 2.26 (0.90, 5.64) | 1.66 (1.01, 2.74) | 1 (ref) |

Model 1: unadjusted

Model 2: adjusted for age, gender, PD vintage, BMI, and comorbidities

Model 3: Model 2 + PD modality and Icodextrin use

**Supplementary Table 2.** Patient characteristics in Japan PDOPPS, overall and by tertile of facility peritonitis rate

| **Patient/treatment characteristics** |  | **Facility Peritonitis rate (episodes/patient-year) tertile** | | |
| --- | --- | --- | --- | --- |
|  | **Overall** | **1 (0.03-0.13)** | **2 (0.14-0.34)** | **3 (0.34-0.50)** |
| **Patients, N** | 1200 | 273 | 469 | 458 |
| **Demographics** |  |  |  |  |
| Age, years | 65 [55,73] | 62 [53,70] | 65 [55,73] | 67 [57,75] |
| Female sex | 33% | 33% | 34% | 33% |
| Time on PD, years | 1.0 [0.0,2.9] | 1.7 [0.2,3.6] | 0.9 [0.0,2.7] | 0.8 [0.0,2.6] |
| Urine volume > 200 mL/day | 83% | 85% | 82% | 84% |
| Body mass index, kg/m^2^ | 23.0 (3.6) | 23.3 (4.0) | 23.1 (3.4) | 22.9 (3.5) |
| Current smoker | 11% | 10% | 11% | 12% |
| **Comorbidities** |  |  |  |  |
| Diabetes | 40% | 34% | 41% | 43% |
| Hypertension | 95% | 95% | 95% | 94% |
| Congestive heart failure | 18% | 14% | 21% | 16% |
| Coronary artery disease | 15% | 11% | 13% | 20% |
| Cerebrovascular disease | 13% | 13% | 14% | 12% |
| Peripheral vascular disease | 7% | 6% | 7% | 7% |
| Other cardiovascular disease | 13% | 7% | 15% | 15% |
| **PD prescription** |  |  |  |  |
| CAPD (vs. APD) | 61% | 44% | 58% | 74% |
| Icodextrin | 44% | 46% | 45% | 43% |
| **Labs** |  |  |  |  |
| Albumin, g/dL | 3.3 (0.5) | 3.3 (0.4) | 3.4 (0.5) | 3.2 (0.5) |
| Hemoglobin, g/dL | 11.0 (1.3) | 11.0 (1.2) | 11.0 (1.3) | 10.9 (1.4) |
| Phosphorus, mg/dL | 5.0 (1.2) | 5.2 (1.4) | 5.0 (1.2) | 4.9 (1.2) |
| Potassium, mEq/L | 4.2 (0.7) | 4.2 (0.6) | 4.3 (0.6) | 4.1 (0.8) |
| Hypokalemia^a^ | 13% | 14% | 7% | 18% |
| **Medications** |  |  |  |  |
| Proton pump inhibitor | 41% | 43% | 42% | 38% |
| H2-blocker | 4% | 3% | 4% | 4% |

Results shown as mean (standard deviation), median [interquartile range], or prevalence

^a^ Defined as a serum potassium level <3.5 mEq/L.

**Supplementary Table 3.** Facility characteristics in Japan PDOPPS, overall and by tertile of facility peritonitis rate

| **Facility characteristic/practice** |  | **Facility peritonitis rate**  **(episodes/patient-year) tertile** | | |
| --- | --- | --- | --- | --- |
|  | **Overall** | **1 (0.03-0.13)** | **2 (0.14-0.34)** | **3 (0.34-0.50)** |
| **Facilities, n** | 48 | 16 | 17 | 15 |
| **Patients and staffing** |  |  |  |  |
| Facility size | 31 [25,42] | 29 [21,39] | 32 [29,40] | 29 [26,81] |
| Patient to physician ratio |  |  |  |  |
| <4 | 23% | 36% | 31% | 0% |
| 4-6 | 20% | 18% | 25% | 15% |
| 7-9 | 33% | 36% | 38% | 23% |
| 10+ | 25% | 9% | 6% | 62% |
| Patient to nurse ratio |  |  |  |  |
| <4 | 35% | 36% | 31% | 38% |
| 4-6 | 15% | 18% | 19% | 8% |
| 7-9 | 38% | 45% | 25% | 46% |
| 10+ | 13% | 0% | 25% | 8% |
| **PD education** |  |  |  |  |
| Total time, hours | 5 [3,10] | 3 [3,5] | 5 [3,10] | 9 [5,10] |
| Training format |  |  |  |  |
| One-on-one | 90% | 100% | 88% | 85% |
| Group | 0% | 0% | 0% | 0% |
| Combination | 10% | 0% | 13% | 15% |
| Timing |  |  |  |  |
| Prior to PD catheter insertion | 60% | 82% | 56% | 46% |
| 1 week after PD catheter insertion | 25% | 18% | 31% | 23% |
| 2+ weeks after PD catheter insertion | 3% | 0% | 6% | 0% |
| Other | 13% | 0% | 6% | 31% |
| Location of initial training |  |  |  |  |
| Home | 0% | 0% | 0% | 0% |
| Facility | 97% | 100% | 94% | 100% |
| Combination | 3% | 0% | 6% | 0% |
| **Antibiotic prophylaxis practice^a^** |  |  |  |  |
| At the time of PD catheter insertion | 90% | 100% | 88% | 78% |
| Routine dental procedures (i.e., cleaning) | 3% | 0% | 6% | 0% |
| Complicated dental procedures (i.e., tooth extraction) | 73% | 86% | 76% | 44% |
| Gynecological procedures (i.e., hysteroscopy, endometrial ablation) | 48% | 57% | 41% | 44% |
| Lower endoscopy (i.e., colonoscopy) | 58% | 79% | 53% | 33% |
| **Screening for S. aureus nasal carriage** |  |  |  |  |
| Yes | 23% | 7% | 29% | 33% |
| No | 78% | 93% | 71% | 67% |
| **Regular medical visits** |  |  |  |  |
| Once a week | 3% | 0% | 0% | 8% |
| Once, every 2-3 weeks | 33% | 18% | 38% | 38% |
| Once a month | 65% | 82% | 63% | 54% |
| **PD/other prescription** |  |  |  |  |
| Facility % icodextrin | 52% [35%,67%] | 56% [38%,72%] | 50% [37%,60%] | 40% [32%,75%] |
| Facility % proton pump inhibitors | 42% [32%,47%] | 39% [21%,54%] | 43% [38%,46%] | 36% [23%,47%] |
| Facility % H2-blocker | 0% [0%,6%] | 0% [0%,3%] | 0% [0%,6%] | 3% [0%,6%] |

Results shown as mean (standard deviation), median [interquartile range], or prevalence

^a^ Procedures/situations where antibiotic prophylaxis is used or recommended for PD patients at your center; Japan PDOPPS medical director survey

**Supplementary Table 4.** Patient characteristics associated with time to first peritonitis; effect of progressive adjustment

| **Patient/treatment characteristics** | **Hazard ratio (95% CI)** | | | | |
| --- | --- | --- | --- | --- | --- |
|  | **Model 1** | **Model 2** | **Model 3** | **Model 4** | **Model 5** |
| **Demographics** |  |  |  |  |  |
| Age, per 5 years | 1.11 (1.05,1.16) | 1.1 (1.04,1.16) | 1.07 (1.00,1.14) | 1.07 (1.01,1.14) | 1.07 (1.01,1.14) |
| Female sex | 0.86 (0.67,1.10) | 0.87 (0.67,1.14) | 0.82 (0.62,1.09) | 0.82 (0.62,1.08) | 0.82 (0.62,1.08) |
| Time on PD, per year | 0.99 (0.95,1.03) | 0.99 (0.95,1.03) | 1.00 (0.96,1.05) | 1.01 (0.96,1.05) | 1.01 (0.96,1.05) |
| Body mass index, per kg/m^2^ | 1.00 (0.97,1.04) | 1.00 (0.97,1.04) | 1.02 (0.98,1.06) | 1.02 (0.98,1.06) | 1.02 (0.98,1.06) |
| Current smoker |  | 1.11 (0.84,1.47) | 1.15 (0.84,1.57) | 1.13 (0.82,1.54) | 1.12 (0.82,1.54) |
| **Comorbidities** |  |  |  |  |  |
| Diabetes |  | 0.98 (0.76,1.27) | 0.92 (0.71,1.19) | 0.92 (0.71,1.19) | 0.92 (0.71,1.20) |
| Hypertension |  | 1.11 (0.71,1.73) | 1.16 (0.76,1.78) | 1.12 (0.73,1.73) | 1.12 (0.73,1.71) |
| Congestive heart failure |  | 0.98 (0.73,1.32) | 1.01 (0.74,1.38) | 1.02 (0.74,1.40) | 1.01 (0.74,1.39) |
| Coronary artery disease |  | 1.03 (0.73,1.46) | 1.01 (0.69,1.47) | 0.96 (0.65,1.42) | 0.96 (0.65,1.42) |
| Cerebrovascular disease |  | 0.86 (0.63,1.17) | 0.80 (0.58,1.11) | 0.79 (0.57,1.09) | 0.79 (0.57,1.10) |
| Peripheral vascular disease |  | 1.13 (0.73,1.74) | 1.17 (0.78,1.74) | 1.16 (0.77,1.74) | 1.16 (0.77,1.73) |
| Other cardiovascular disease |  | 1.28 (0.91,1.81) | 1.16 (0.85,1.58) | 1.17 (0.87,1.59) | 1.17 (0.87,1.59) |
| **PD prescription** |  |  |  |  |  |
| CAPD (vs. APD) |  |  | 1.34 (1.08,1.67) | 1.35 (1.09,1.67) | 1.34 (1.09,1.66) |
| Icodextrin |  |  | 0.86 (0.68,1.09) | 0.85 (0.68,1.08) | 0.85 (0.68,1.08) |
| **Labs** |  |  |  |  |  |
| Albumin, per g/dL |  |  | 0.61 (0.46,0.80) | 0.60 (0.46,0.80) | 0.60 (0.46,0.80) |
| Hemoglobin, per g/dL |  |  | 0.99 (0.89,1.09) | 0.99 (0.90,1.09) | 0.99 (0.90,1.09) |
| Phosphorus, per mg/dL |  |  | 0.94 (0.85,1.04) | 0.95 (0.85,1.05) | 0.95 (0.85,1.05) |
| Potassium, mEq/L |  |  |  |  |  |
| <3.5 |  |  | 1.11 (0.80,1.56) | 1.11 (0.79,1.55) | 1.10 (0.79,1.54) |
| 3.5 – 5 |  |  | 1 (ref) | 1 (ref) | 1 (ref) |
| >5 |  |  | 1.45 (0.99,2.11) | 1.41 (0.95,2.10) | 1.41 (0.95,2.09) |
| **Medications** |  |  |  |  |  |
| Proton pump inhibitor |  |  |  | 1.11 (0.88,1.40) |  |
| H2 blocker |  |  |  | 1.33 (0.56,3.14) |  |
| Any gastric acid suppressant |  |  |  |  | 1.13 (0.88,1.45) |

Number of patients: 1225; number of events: 364

Model 1: individual characteristics only.

Model 2: Model 1 + current smoking status, and 7 comorbidities.

Model 3: Model 2 + PD prescription (i.e., PD type, icodextrin use) and labs (i.e., albumin, hemoglobin, phosphorus, and potassium).

Model 4: Model 3 + medications (i.e., proton pump inhibitor and H2 blocker) use.

Model 5: Model 3 + any gastric acid suppressant use

**Supplementary Table 5.** Facility characteristics/practices associated with patient time to first peritonitis

|  | **Hazard Ratio (95% CI)** | |
| --- | --- | --- |
| **Facility characteristic/practice** | **Unadjusted** | **Case-mix adjusted^a^** |
| **Patients and staffing** |  |  |
| Facility size |  |  |
| Small (<23) | 0.88 (0.60,1.30) | 0.86 (0.60,1.22) |
| Medium (23-41) | 1 (ref) | 1 (ref) |
| Large (>41) | 1.22 (0.82,1.83) | 1.19 (0.83,1.71) |
| Patient to physician ratio |  |  |
| <4 | 0.67 (0.41,1.09) | 0.69 (0.45,1.06) |
| 4-6 | 1 (ref) | 1 (ref) |
| 7-9 | 0.89 (0.55,1.44) | 0.95 (0.64,1.41) |
| 10+ | 1.30 (0.80,2.10) | 1.31 (0.87,1.97) |
| Patient to nurse ratio |  |  |
| <4 | 1.23 (0.83,1.83) | 1.14 (0.76,1.71) |
| 4-6 | 1 (ref) | 1 (ref) |
| 7-9 | 1.21 (0.79,1.86) | 1.18 (0.78,1.78) |
| 10+ | 1.43 (0.73,2.78) | 1.32 (0.65,2.68) |
| **PD education** |  |  |
| Total time, hours |  |  |
| 3-10 | 1 (ref) | 1 (ref) |
| >10 | 1.02 (0.70,1.49) | 1.06 (0.70,1.60) |
| Timing |  |  |
| Prior to PD catheter insertion | 0.83 (0.58,1.20) | 0.87 (0.63,1.20) |
| 1 or 2 weeks after PD catheter insertion | 1 (ref) | 1 (ref) |
| Other | 1.23 (0.95,1.59) | 1.22 (0.93,1.60) |
| **Antibiotic prophylaxis practice,** yes vs no |  |  |
| At the time of PD catheter insertion | 0.61 (0.49,0.77) | 0.63 (0.51,0.78) |
| Complicated dental procedures (i.e., tooth extraction) | 0.73 (0.54,0.98) | 0.74 (0.57,0.95) |
| Gynecological procedures^†^ (i.e., hysteroscopy, endometrial ablation) | 1.00 (0.62, 1.63) | 0.98 (0.61, 1.58) |
| Lower endoscopy (i.e., colonoscopy) | 0.66 (0.50,0.86) | 0.69 (0.53,0.88) |
| **Screening for S. aureus nasal carriage** |  |  |
| Yes vs no | 1.14 (0.82,1.59) | 1.03 (0.78,1.37) |
| **Number of medical visits** |  |  |
| Once a month vs more often than once a month | 0.74 (0.54,1.01) | 0.75 (0.58,0.98) |
| **PD/other prescription** |  |  |
| Facility % icodextrin, per % | 0.76 (0.29,2.04) | 0.76 (0.32,1.82) |
| Facility % proton pump inhibitors, per % | 1.01 (0.28,3.64) | 1.15 (0.39,3.38) |

Number of patients: 1225; number of events: 364

^a^ Adjusted for patient age, sex, time on PD, diabetes, congestive heart failure, and CAPD

^†^ restricted to female patients

**Supplementary Information 2**.

**Facility characteristics by frequency of regular medical visits**

|  | frequency of regular medical visits | | | |
| --- | --- | --- | --- | --- |
|  | Once a week | More often than once a month | Once a month |  |
| Facilities, n | 1 | 13 | 26 |  |
| Patients and staffing |  |  |  |  |
| Facility size | 30 | 40 [31,81] | 28 [23,33] |  |
| Patient to physician ratio |  |  |  |  |
| <4 | 0% | 15% | 27% |  |
| 4-6 | 0% | 0% | 31% |  |
| 7-9 | 0% | 54% | 23% |  |
| 10+ | 100% | 31% | 19% |  |
| Patient to nurse ratio |  |  |  |  |
| <4 | 100% | 31% | 35% |  |
| 4-6 | 0% | 0% | 23% |  |
| 7-9 | 0% | 54% | 31% |  |
| 10+ | 0% | 15% | 12% |  |
| Outcome |  |  |  |  |
| Peritonitis rate, episodes/patient-year | 0.40 | 0.28  [0.18,0.35] | 0.20 [0.13,0.35] |  |
| Episodes with concomitant exit site infection, % | 0% | 0% [0%,11%] | 0% [0%,10%] |  |
